# The role of lysosomes as intermediates in betacoronavirus PHEV egress from nerve cells

**DOI:** 10.1128/jvi.01338-23

**Published:** 2023-11-27

**Authors:** Zhenzhen Wang, Wenqi He, Caili Li, Yuzhu Chen, Zi Li, Yubo Jiao, Jing Zhang, Junchao Shi, Gaili Wang, Jiyu Guan, Kui Zhao, Deguang Song, Feng Gao, Yungang Lan

**Affiliations:** 1 State Key Laboratory for Diagnosis and Treatment of Severe Zoonotic Infectious Diseases, Key Laboratory for Zoonosis Research of the Ministry of Education, Institute of Zoonosis, and College of Veterinary Medicine, Jilin University, Changchun, Jilin, China; 2 Jilin Academy of Animal Husbandry and Veterinary Medicine, Changchun, Jilin, China; Loyola University Chicago - Health Sciences Campus, Maywood, Illinois, USA

**Keywords:** PHEV, virus egress, betacoronaviruses, lysosome, V-ATPase, CNS

## Abstract

The traditional view that betacoronaviruses exit occurs *via* the constitutive secretory route has recently been questioned by studies suggesting that this process involves lysosomal exocytosis. Here, we demonstrated that porcine hemagglutinating encephalomyelitis virus (PHEV), a neurotropic betacoronavirus, traffics to lysosomes prior to engaging in Arl8b-dependent lysosomal exocytosis. Notably, PHEV induces active or passive lysosomal acidification and activates lysosomal degradative enzymes. PHEV release depends on vacuolar H^+^-ATPase-mediated lysosomal acidification. In addition, PHEV transmission and PHEV-induced brain damage in the central nervous system can be blocked by the Rab7 GTPase competitive inhibitor CID1067700. Taken together, lysosome plays a critical role in PHEV egress *in vitro* and *in vivo*.

**IMPORTANCE:**

Betacoronaviruses, including severe acute respiratory syndrome coronavirus-2 (SARS-CoV-2) and mouse hepatitis virus (MHV), exploit the lysosomal exocytosis pathway for egress. However, whether all betacoronaviruses members use the same pathway to exit cells remains unknown. Here, we demonstrated that porcine hemagglutinating encephalomyelitis virus (PHEV) egress occurs by Arl8b-dependent lysosomal exocytosis, a cellular egress mechanism shared by SARS-CoV-2 and MHV. Notably, PHEV acidifies lysosomes and activates lysosomal degradative enzymes, while SARS-CoV-2 and MHV deacidify lysosomes and limit the activation of lysosomal degradative enzymes. In addition, PHEV release depends on V-ATPase-mediated lysosomal pH. Furthermore, this is the first study to evaluate βCoV using lysosome for spreading through the body, and we have found that lysosome played a critical role in PHEV neural transmission and brain damage caused by virus infection in the central nervous system. Taken together, different betacoronaviruses could disrupt lysosomal function differently to exit cells.

## INTRODUCTION

Coronaviruses (CoVs) are members of a large family of enveloped, positive-strand RNA viruses that possess club-like spikes on virion surfaces, single-stranded RNA genomes, and a unique replication strategy (1). Phylogenetic analysis has revealed that CoV family members fall into four major genera that include alpha (α), beta (β), gamma (γ), and delta (Δ) CoVs. Importantly, betacoronaviruses (βCoVs) evolve rapidly due to the genetic flexibility that has enabled them to infect myriad mammalian hosts, such as humans, pigs, cats, bats, civets, dogs, and camels. βCoVs have caused considerable human and livestock morbidity and mortality, serious public health issues, and economic losses. Indeed, human populations have been threatened by recent pandemics of severe respiratory illnesses caused by three βCoVs: severe acute respiratory syndrome coronavirus (SARS-CoV), middle east respiratory syndrome coronavirus, and severe acute respiratory syndrome coronavirus-2 (SARS-CoV-2).

Porcine hemagglutinating encephalomyelitis virus (PHEV）is a βCoV that was first isolated in 1962 in Canada from suckling piglets with encephalomyelitis (2, 3). At that time, PHEV was the only neurotropic porcine CoV known to cause neurological damage and high mortality (2, 3). PHEV also displays neurotropism in mice and rats and produces acute encephalomyelitis. The results of previous *in vivo* or *in vitro* studies using mice or mouse neuroblastoma (N2a) cells have suggested that PHEV invades the central nervous system (CNS) *via* the peripheral nervous system (i.e., neural spread) and infects nerve cells (4). Shi et al. found that PHEV could invade the CNS *via* the olfactory and trigeminal nerves by using intranasal inoculation of BALB/c mice with PHEV (5). Herein, we demonstrate that PHEV egress from nerve cells involves the viral hijacking of cellular lysosomal trafficking pathways, and lysosome plays a critical role in PHEV transmission *in vivo*. Mechanistically, PHEV-hijacked lysosomes were found to be actively or passively acidified, which supported increased activities of lysosomal proteases, such as cathepsin D (CTSD) and cathepsin B (CTSB), with lysosomal acidification found to play a key role in the overall PHEV cellular exit strategy. Notably, this mechanism differs from corresponding cellular egress mechanisms used by mouse hepatitis virus (MHV) and SARS-CoV-2, which depend on a deacidified lysosomal environment (6, 7).

## RESULTS

### PHEV is enriched in late endosomes/lysosomes during replication

**Fig 1:**
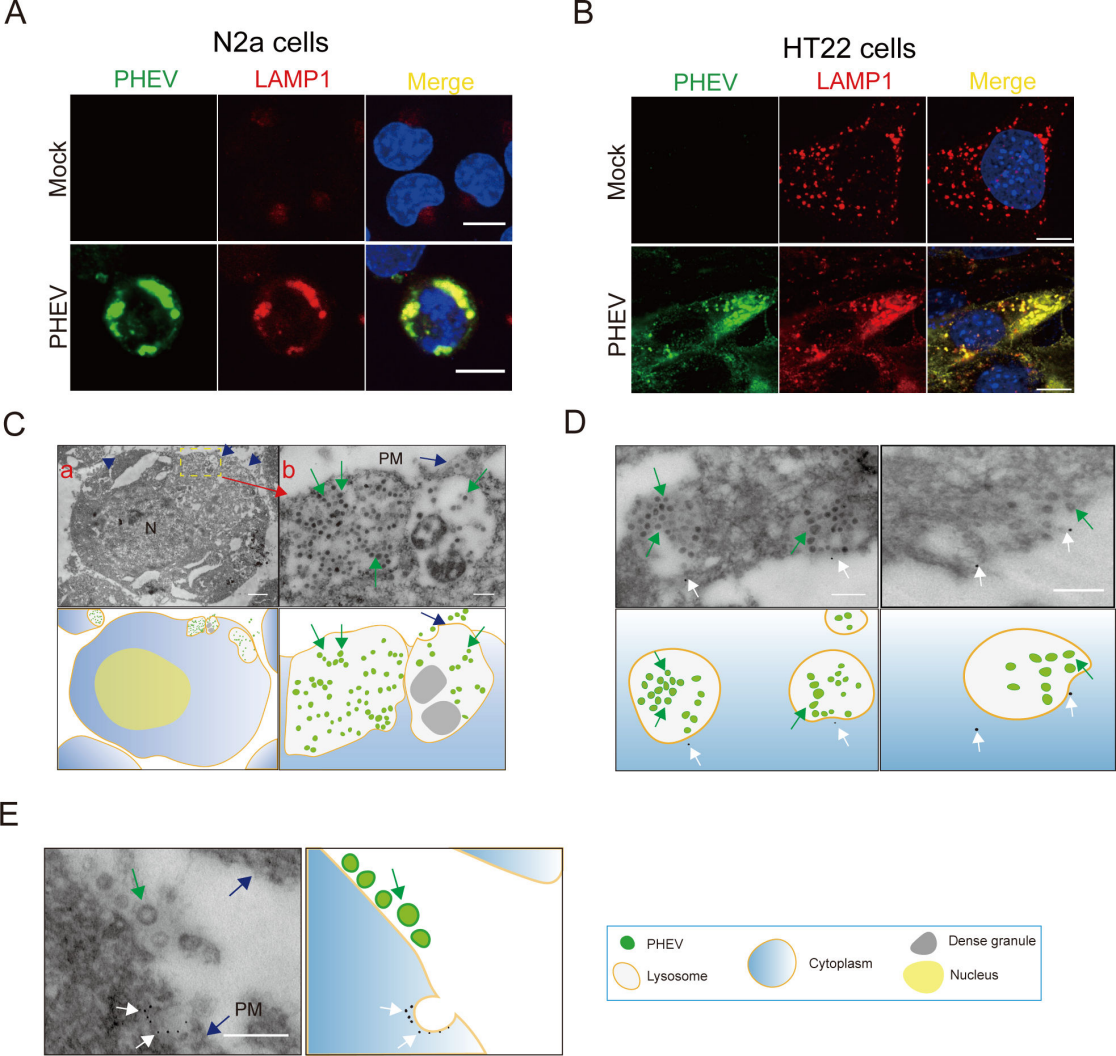
PHEV is enriched in late endosomes/lysosomes during replication. (**A and B**) Colocalization of PHEV and LAMP1 in N2a and HT22 cells, respectively. Mock- and PHEV-infected cells were immunostained with anti-LAMP1 (red) and anti-PHEV (green) antibodies at 48 hpi. Scale bar, 10 µm. (**C**) TEM images of PHEV-infected N2a cells at 48 hpi. Blue arrows: The plasma membrane; Green arrows: PHEV. Scale bar, 1 µm (**A**) and 200 nm (**B**). (**D and E**) PHEV-infected N2a cells at 48 hpi were processed for immuno-EM and labeled with anti-LAMP1 and 12 nm colloidal gold. Blue arrows: the plasma membrane; White arrows: the colloidal gold-labeled LAMP1; Green arrows: PHEV. Scale bar, 200 nm. Representative images are shown.

Indeed, virus particles in the cytoplasm of PHEV-infected cells were found to contain in groups within vesicles and lysosome-like structures, which were often observed in the Golgi areas and beneath the cell surface by ultrastructurally examining PHEV-infected rat dorsal root ganglia in previous reports (8). However, whether lysosome-like structures containing PHEV particles are lysosomes and PHEV release from nerve cells is dependent on the lysosomal exocytic pathway needs to be determined. To confirm that lysosome-like structures containing PHEV particles were lysosomes and that PHEV release from nerve cells depends on lysosomal exocytic pathway function, we first researched PHEV replication kinetics and cellular egress using N2a cells or hippocampal neurons (HT22 cells). Our results revealed that the greatest rate of PHEV egress and a large number of infectious viral particles were observed at 24–48 h post-infection (hpi) and then gradually plateaued (Fig. S1A through D). Notably, viral egress at 48 h took place in the absence of any cell lysis because there was no significant change in the permeability of the plasma membrane by measuring the uptake of the membrane-impermeant dye trypan blue and propidium iodide (Fig. S1E and F). The productive phase of infectious viral particle release at 48 hpi apparently proceeded without any obviously detectable cell damage, and PHEV-infected N2a or HT22 cells at 60 hpi appeared rather uniform in evenly spread rounding and sloughing of cells throughout the cell culture flask (Fig. S1G and H). Subsequently, we investigated the lysosomal distribution of PHEV by exploring the colocalization of PHEV nucleocapsid (N) protein with lysosomal-associated membrane protein 1 (LAMP1) at 48 hpi. For the results, we found that PHEV localized to lysosomes (Fig. 1A and B) (4), and LAMP1 did not colocalize with markers of the Golgi apparatus and endoplasmic reticulum (ER; Fig. S2A and B). In addition, we found that PHEV could colocalize with LAMP1-mcherry, an artificial reporter that labeled late endosomes and lysosomes in cells (Fig. S2C), and LAMP1-mcherry did not colocalize with markers of the Golgi apparatus and endoplasmic reticulum (data not shown). By using transmission electron microscopy (TEM), we found that all virus particles were present within large lysosome/late endosome-like structures at 48 hpi (Fig. 1C). Furthermore, these cellular structures were confirmed to be lysosomes/late endosomes using an immunoelectron microscopy-based methodology (immuno-EM) to quantitatively analyze LAMP1 and virus particle distributions (Fig. 1D and E). Notably, immuno-EM results revealed that in infected cells, some LAMP1^+^/PHEV^+^ organelles were located just beneath the plasma membrane (Fig. 1E, blue arrows), with LAMP1 found to be present on the internal plasma membrane surface (Fig. 1E, white arrows). This result suggested that the significant fusion of lysosomes with plasma membranes occurred during PHEV release from cells.

### CID1067700 inhibits PHEV egress

**Fig 2:**
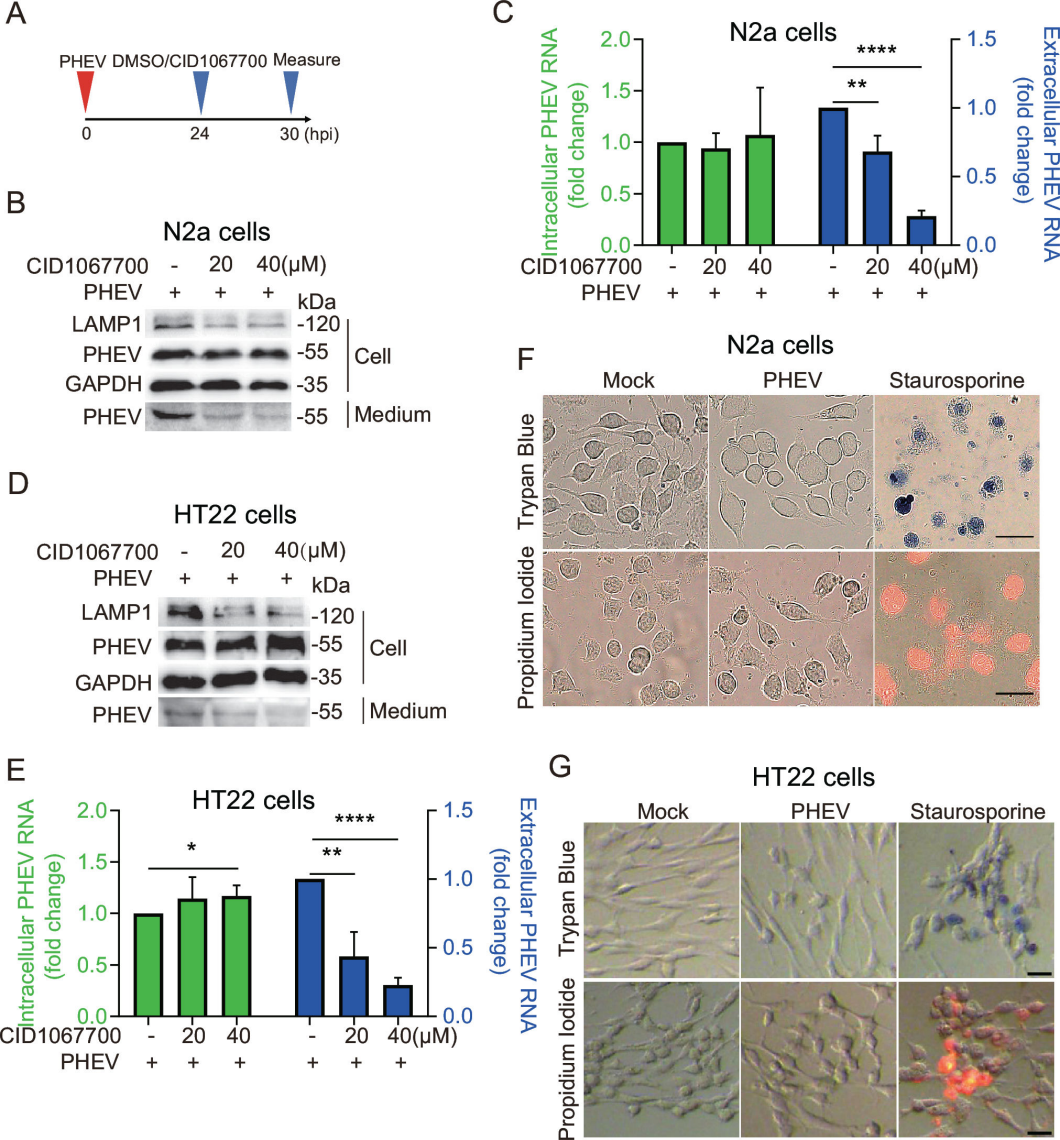
PHEV utilizes late endosomes/lysosomes for egress. (**A**) Schematic diagrams illustrating the experimental design for time-of-addition experiments. (**B**) The protein levels of LAMP1, PHEV, and glyceraldehyde-3-phosphate dehydrogenase (GAPDH) were analyzed by western blot in PHEV-infected N2a cells treated with CID1067700 or DMSO. (**C**) The PHEV N genomic RNA was determined using quantitative PCR (qPCR) in DMSO- or CID1067700-treated PHEV-infected N2a cells. The data were normalized to the DMSO-treated PHEV-infected cells. (**D**) The protein levels of LAMP1, PHEV, and GAPDH were analyzed by western blot in PHEV-infected HT22 cells treated with CID1067700 or DMSO, respectively. (**E**) The PHEV N genomic RNA was determined using qPCR in DMSO- or CID1067700-treated PHEV-infected HT22 cells. The data were normalized to the DMSO-treated PHEV-infected cells. (**F and G**) Trypan blue and propidium iodine exclusion were used to detect changes in plasma membrane permeability in PHEV-infected N2a or HT22 cells at 30 hpi. Staurosporine-treated cells were seen as a positive control of cell membrane rupture. Scale bar, 30 µm. Representative blots and images are shown. Data are shown as mean ± SD. *P* values were considered significant when *P* < 0.05 and denoted as, *, *P* < 0.05, **, *P* < 0.01, and ****, *P* < 0.0001.

Rab7, a small GTPase belonging to the Rab family, localizes to lysosomes, late endosomes, and multivesicular bodies (9, 10). There the enzyme plays critical roles in lysosome biogenesis and lysosome maintenance, as evidenced by the suppressive effects of Rab7 depletion on the maturation of late endosomes and multivesicular bodies that leads to reduced lysosome numbers (7, 11). In order to investigate the effects of reduced Rab7 activity or lysosome numbers on PHEV egress, PHEV-infected N2a cells or HT22 cells were treated with CID1067700 for 6 h after 24 h infection with PHEV (Fig. 2A). The results revealed that the addition of inhibitor to cells did not affect cell viability (data not shown) or intracellular PHEV load in N2a cells (Fig. 2B). However, the inhibitor potently reduced viral egress in a dose-dependent manner, with ~30% and ~80% reductions in virus egress observed for 20 and 40 µM concentrations of CID1067700 (Fig. 2B and C). Moreover, we also found that CID1067700 treatment significantly inhibited PHEV egress from HT22 cells (Fig. 2D and E). Notably, viral egress took place in the absence of any cell lysis under our experimental design (Fig. 2A) because there was no significant change in the permeability of the plasma membrane by measuring the uptake of the membrane-impermeant dye trypan blue and propidium iodide (Fig. 2F and G). Furthermore, we observed a ~50% decrease in LAMP1 protein level (Fig. 2B and D) and LAMP1^+^ punctate organelles (per cell; Fig. S2D), suggesting a decrease in lysosome numbers. Thus, these results further support a critical regulatory role of lysosomal biogenesis in PHEV egress from cells.

### PHEV uses an Arl8b-dependent lysosomal exocytic pathway for cellular egress

**Fig 3:**
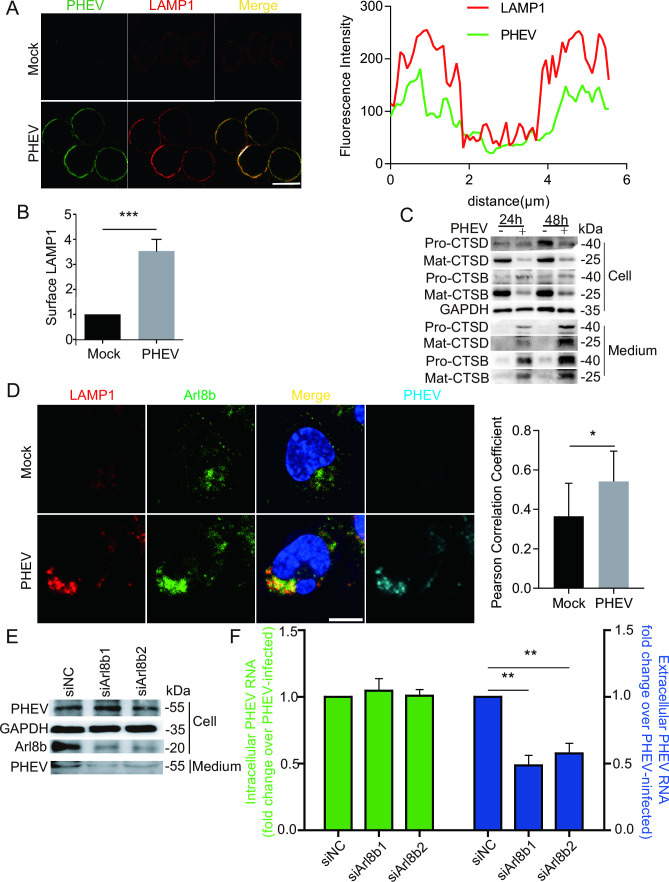
PHEV uses an Arl8b-dependent lysosomal exocytic pathway to egress. (**A**) Colocalization of PHEV and surface LAMP1. Mock- or PHEV-infected cells at 48 hpi were immunostained with anti-LAMP1 (red) and anti-PHEV (green) antibodies. Scale bar, 10 µm. (**B**) The surface LAMP1 levels on mock- and PHEV-infected cells from the experiment whose results are shown in panel A were quantified by using Image J. (**C**) The protein levels of Pro-CTSD, Pro-CTSB, Mat-CTSD, Mat-CTSB, and GAPDH at 24 and 48 hpi were analyzed by western blot, respectively. (**D**) Mock- or PHEV-infected cells at 48 hpi were immunostained with anti-LAMP1 (red), anti-Arl8b (green), and anti-PHEV (teal) antibodies. Scale bar, 10 µm. (**E**) The protein levels of Arl8b, PHEV, and GAPDH were analyzed by western blot in PHEV-infected Arl8b small interfering RNA (siRNA)-treated cells or PHEV-infected-non-target siRNA-treated cells. (**F**) The PHEV N genomic RNA was determined using qPCR in Arl8b siRNA-treated cells and non-target siRNA-treated cells. The data were normalized to the non-target siRNA-treated cells. Representative blots and images are shown. Data are shown as mean ± SD. *P* values were considered significant when *P* < 0.05 and denoted as, *, *P* < 0.05, **, *P* < 0.01, and ***, *P* < 0.001.

Lysosomal exocytosis is a pathway whereby lysosomes traffic to the cell periphery and fuse with the plasma membrane to release their luminal contents to the external environment (12). We found that levels of plasma membrane LAMP1, a protein involved in lysosomal exocytosis, were ~3.5-fold higher in infected cells as compared to levels in mock-infected cells at 48 hpi (Fig. 3A and B). In addition, we measured the levels of CTSD and CTSB and their precursor proteins Pro-CTSD and Pro-CTSB, respectively, and their mature forms produced by cleavage by lysosomal proteases, Mat-CTSD and Mat-CTSB, respectively. Collection of extracellular media from PHEV-infected cells revealed gradually increasing levels of secreted Mat-CTSD and Mat-CTSB (Fig. 3C), indicating that PHEV infection induced lysosomal exocytosis.

Arl8b, a small Arf-like GTPase belonging to the Ras family, localizes to late endosomes/lysosomes and regulates their movement to the plasma membrane as a key step in exocytosis (7, 13, 14). To investigate the potential role of Arl8b in PHEV release, cells were transiently transfected with Arl8b-GFP and then were infected with PHEV for 24 h, followed by immunostaining with anti-PHEV and anti-LAMP1 antibodies. The results revealed the detection of numerous Arl8b^+^/PHEV^+^/LAMP1^+^ organelles (Fig. S2E), thus indicating that exogenous Arl8b was recruited to lysosomes during PHEV release. In addition, mock or PHEV-infected cells at 48 hpi were immunostained with anti-Arl8b, anti-LAMP1, and anti-PHEV antibodies. The results revealed PHEV infection led to the enhancement colocalization of endogenous Arl8b and LAMP1 (Fig. 3D). Moreover, after cells were treated for 24 h with small interfering RNAs (siRNAs), including Arl8b-targeting and non-targeting control siRNAs, cells were infected with PHEV for 24 h and then extracellular supernatants and cell lysates were collected. Results obtained *via* analysis of protein levels in cell lysates revealed ~70% downregulation of Arl8b protein in Arl8b siRNA-treated cells (Fig. 3E). Furthermore, quantitative PCR (qPCR) measurements of genomic PHEV N RNA levels in cell supernatants demonstrated a ~50% reduction in viral genome release by Arl8b siRNA-treated cells as compared with non-target siRNA-treated cells, whereas intracellular PHEV protein and RNA expression levels were unaffected (Fig. 3E and F). Taken together, these results confirmed that PHEV utilizes Arl8b-dependent lysosomal exocytic pathways for egress during infection.

### PHEV hijacks actively or passively acidified lysosomes to exit cells

**Fig 4:**
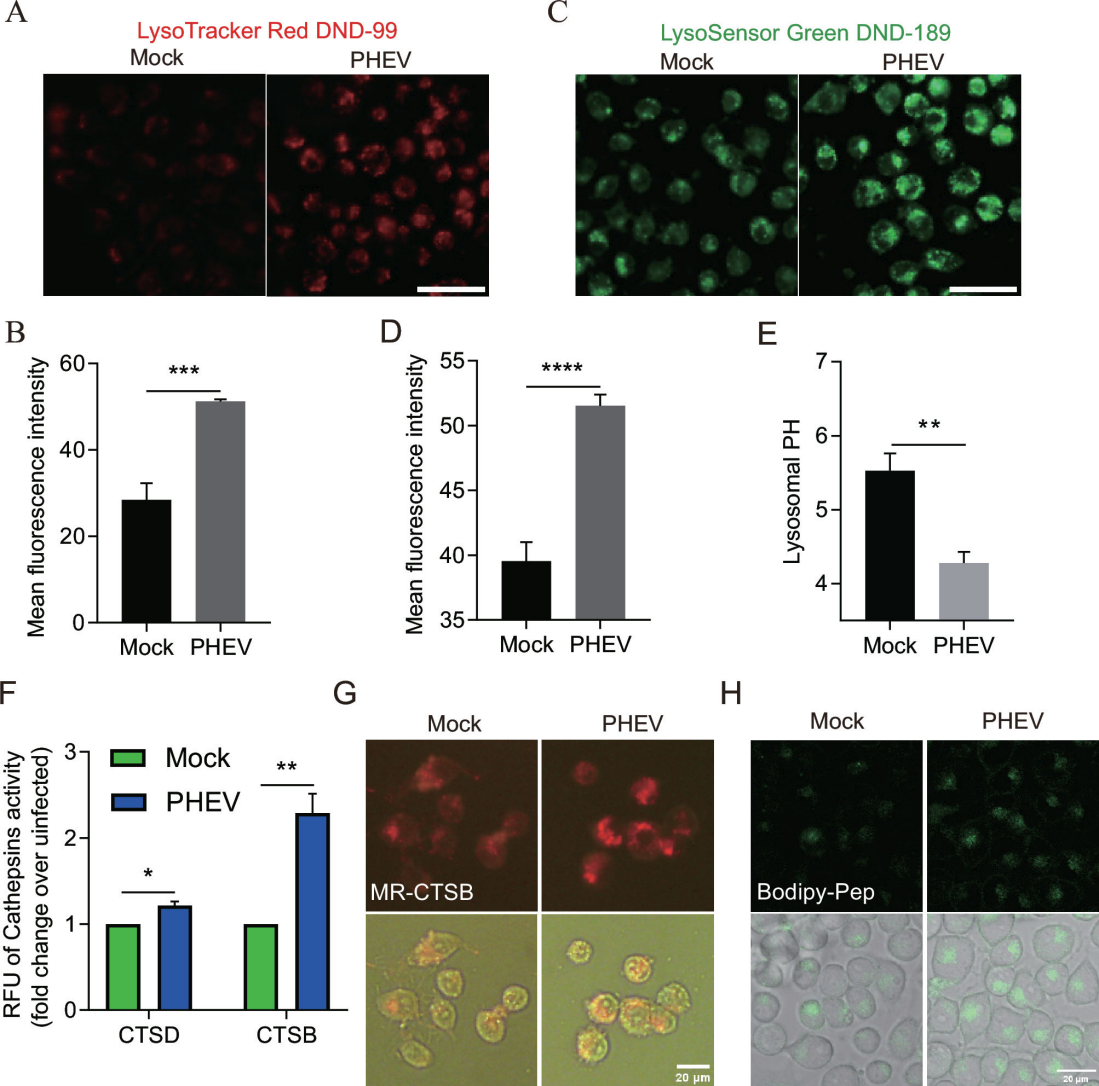
PHEV hijacked actively or passively acidified lysosomes and increased lysosomal degradation enzyme activation. (**A**) Mock or PHEV-infected N2a cells at 48 hpi were stained by LysoTracker Red DND-99. Scale bar, 10 µm. (**B**) Quantification of LysoTracker Red DND-99 fluorescence intensity. (**C**) Mock- or PHEV-infected N2a cells at 48 h were stained by LysoSensor green DND-189. Scale bar, 10 µm. (**D**) Quantification of LysoSensor Green DND-189 intensity. (**E**) Mean LysoSensor Green fluorescence intensity in mock- and PHEV-infected cell groups converted to a pH value from calibration of the dye. (**F**) Lysosome enzyme (CTSD and CTSB) activity of mock- and PHEV-infected N2a cells at 48 hpi. (**G and H**) Live imaging of Magic Red and Bodipy-FL-pepstatin A dye in N2a cells, which were used as indirect indicators of CTSB and CTSD enzyme activities, respectively. Representative images are shown. Data are shown as mean ± SD. *P* values were considered significant when *P* < 0.05 and denoted as, *, *P* < 0.05, **, *P* < 0.01, ***, *P* < 0.001, and ****, *P* < 0.0001.

Normally, the acidic lysosomal environment helps destroy invading viruses and other pathogens before they can leave cells to start another infection cycle. To assess the functional consequences of the PHEV egress pathway on lysosomal acidity, N2a cells infected with PHEV for 48 h were labeled with LysoTracker Red DND-99, a cell-permeable weakly basic dye that is acidotropic (accumulates in acidified organelles). We observed the mean fluorescence intensity was markedly increased, indicating that increases in both acidity and number of acidified lysosomes occurred in PHEV-infected cells as compared with mock-infected cells (Fig. 4A and B). To further investigate the effect of PHEV infection on lysosomal pH, we quantified the lysosomal pH of infected cells using LysoSensor Green DND-189, a dye that can accumulate in acidified organelles. Importantly, due to the low pK of this dye (5.2), this dye only fluoresces when it is present within highly acidic organelles (e.g., lysosomes), whereby its fluorescence amplitude changes with pH in a calibratable manner. Using this dye, we found that the mean pH of lysosomes in mock-infected cells was 5.524 (within the pH range of 5.258–5.717), while in PHEV-infected cells, the pH was 4.087 (within the pH range of 3.514–4.394; Fig. 4C through E). These results revealed that highly significant acidification increases in lysosomes of infected N2a cells, as reflected by pH values that were one full pH unit lower than corresponding values obtained for mock-infected cells. Moreover, both the mean fluorescence intensity of LysoTracker and LysoSensor were elevated significantly in porcine kidney epithelial cells (PK-15 cells) and mouse primary neurons after PHEV infection (data not shown), further demonstrating that PHEV infection broadly induced active or passive lysosome acidification of host cells.

Lysosomes are the primary degradative compartment of the cell that helps destroy viruses and other pathogens by the resident hydrolytic enzymes activated by the highly acidic pH within the lysosomal lumen (15). Interestingly, the collection of PHEV-infected cells at 48 hpi revealed increasing activation of CTSD and CTSB proteases *via* CTSD and CTSB activity detection (Fig. 4F). Additionally, we used cell-permeant Magic Red and Bodipy-pepstatin A to measure the activity of CTSB and CTSD, respectively. Results showed that PHEV-infected N2a cells had higher fluorescence intensity of Magic Red and Bodipy-pepstatin A (Fig. 4G and H), indicating that PHEV infection increased CTSB and CTSD activity. Taken together, these results suggested that PHEV hijacked actively or passively acidified lysosomes and increased the activation of lysosomal proteases.

### PHEV release depends on V-ATPase-dependent lysosomal acidification

The above results showed that PHEV infection led to active or passive acidification of lysosomes. In addition, the acidification would likely enhance lysosomal protease activities that would degrade the virus as a prediction that is inconsistent with the observed successful egress of intact virions, prompting us to examine the relationship between PHEV infection and the acidic environment within lysosomes.

**Fig 5:**
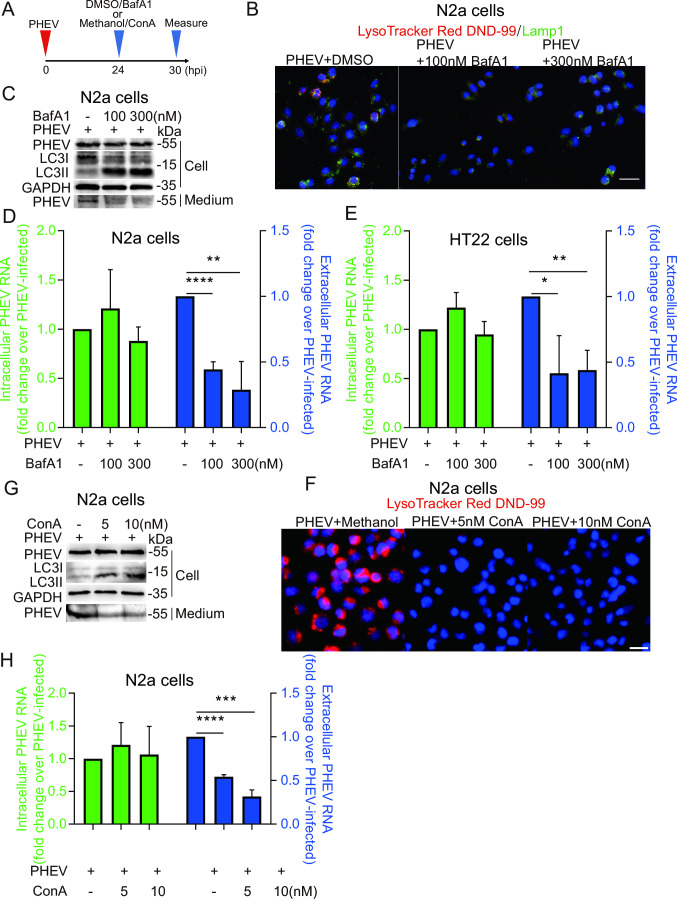
PHEV egress in a lysosomal pH-dependent manner. (**A**) Schematic diagrams illustrating the experimental design for time-of-addition experiments. (**B**) The PHEV-infected N2a cells treated with BafA1 or DMSO were immunostained with LysoTracker Red DND-99 (red) and LAMP1 (green). Scale bar, 20 µm. (**C**) The protein levels of LC3, PHEV, and GAPDH were analyzed by western blot in DMSO- or BafA1-treated PHEV-infected N2a cells. (**D and E**) The PHEV N genomic RNA was determined using qPCR in DMSO- or BafA1-treated PHEV-infected N2a and HT22 cells, respectively. The data were normalized to the DMSO-treated PHEV-infected cells. (**F**) The protein levels of LC3, PHEV, and GAPDH were analyzed by western blot in Methanol- or ConA -treated PHEV-infected N2a cells. (**G**) The PHEV-infected N2a cells treated with ConA or Methanol were stained with LysoTracker Red DND-99. Scale bar, 20 µm. (**H**) The PHEV N genomic RNA was determined using qPCR in Methanol- or ConA-treated PHEV-infected N2a cells. The data were normalized to the methanol-treated PHEV-infected cells. Representative blots and images are shown. Data are shown as mean ± SD. *P* values were considered significant when *P* < 0.05 and denoted as, *, *P* < 0.05, **, *P* < 0.01, ***, *P* < 0.001, and ****, *P* < 0.0001.

The low internal pH of lysosomes is maintained *via* proton pumps acting through multiple types of membrane channels, with most protons transported to lysosomal lumens through vacuolar H^+^ ATPase (V-ATPase)-associated proton channels (16). To further investigate the relationship between lysosomal pH and PHEV egress, PHEV-infected N2a cells were treated with the V-ATPase inhibitor bafilomycin A1 (BafA1) for 6 h after 24 h infection with PHEV (Fig. 5A). We found that both 100 and 300 nM BafA1 treatments led to significantly reduced numbers of LysoTracker Red^+^ puncta (Fig. 5B). Moreover, at 30 hpi, although BafA1 treatment inhibited lysosomal degradation of microtubule-associated protein 1A/1B-light chain 3-II proteins (LC3-II) without affecting intracellular PHEV N-protein expression levels and RNA copy numbers, extracellular levels of these indicators rapidly decreased after the addition of the inhibitor to cells (Fig. 5C and D). Similarly, we found that BafA1 treatment inhibited PHEV release in HT22 cells (Fig. 5E), and another specific V-ATPase inhibitor concanamycin A (ConA) significantly inhibited PHEV-induced lysosome acidification and decreased extracellular PHEV N-protein expression levels and RNA copy numbers in a dose-dependent manner (Fig. 5F through H). However, lysosomotropic chloroquine (CQ) could not significantly inhibit PHEV-induced lysosome acidification by releasing basic side chains that raise intralysosomal pH under our experimental design like BafA1 or ConA treatment, and then, the reduction of PHEV release could not be observed (Fig. S3A and B). Taken together, these results indicate that PHEV release depends on V-ATPase-dependent lysosomal acidification.

### The PHEV release mechanism involves disorders of V0a3-dependent lysosomal acidification

**Fig 6:**
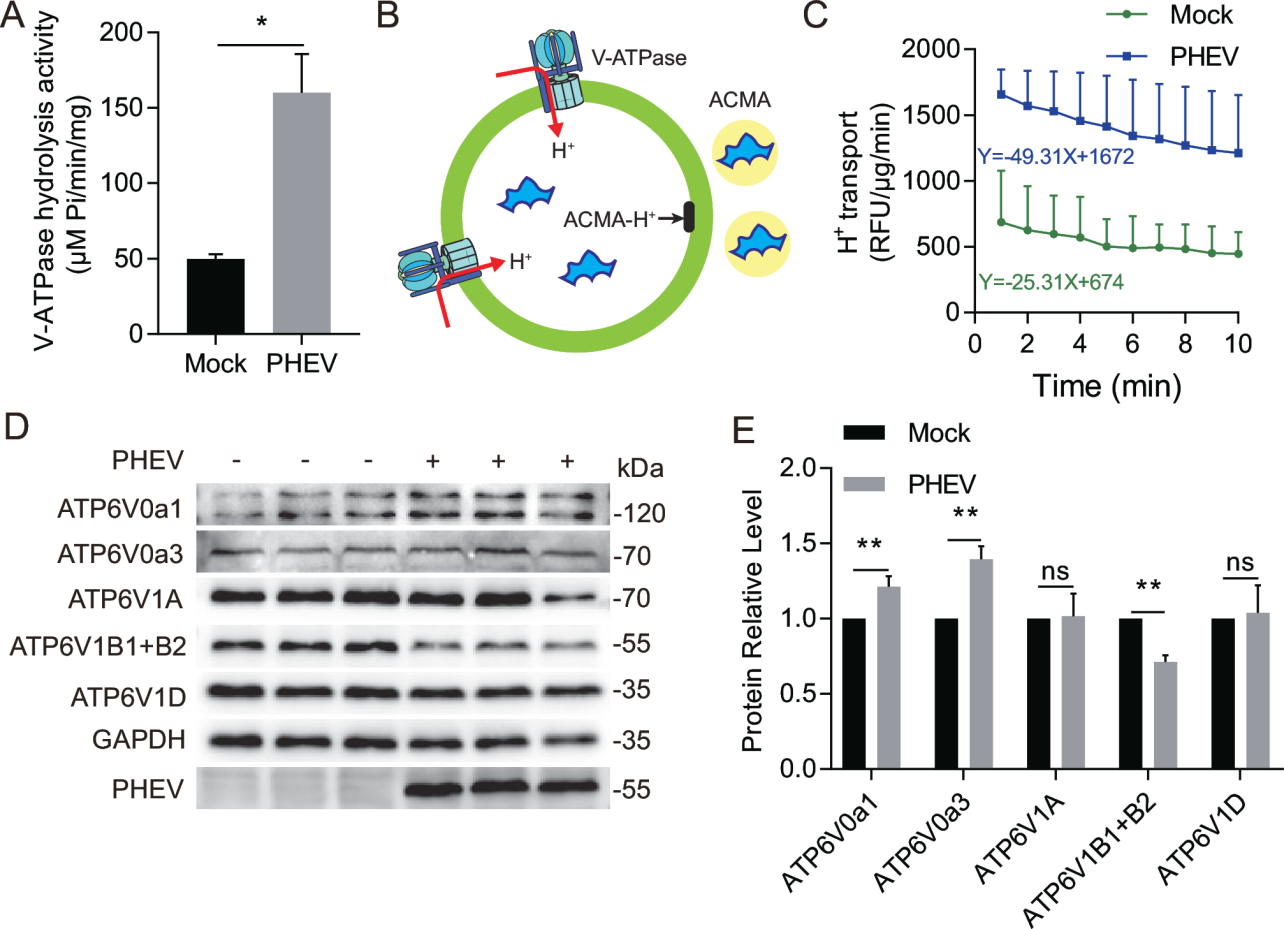
PHEV infection leads to the enhancement of lysosomal V-ATPase activity. (**A**) The V-ATPase hydrolytic activity in mock- or PHEV-hijacked purified lysosomes at 48 hpi. (**B**) Schematic of the ACMA assay. A lipid-soluble proton-sensitive dye (ACMA) is permeated to lysosomes (green circle). Protons moving into the lysosomes via V-ATPase bind ACMA to form ACMA-H+, which does not fluoresce, resulting in a decrease in total fluorescence emission. Protonated ACMA cannot pass out through the lysosomal membrane. (**C**) The proton translocation ability of V-ATPase in mock- and PHEV-hijacked purified lysosomes at 48 hpi. (**D**) The protein levels of various V-ATPase subunits in mock- and PHEV-infected cells at 48 hpi. (**E**) The ratios of the intensity values of the V-ATPase/GAPDH immunoblotting results from the experiment whose results are shown in panel D were quantified by using Image J. Data are shown as mean ± SD. *P* values were considered significant when *P* < 0.05 and denoted as, *, *P* < 0.05, **, *P* < 0.01.

In order to further confirm the role of V-ATPase in PHEV-induced lysosomal acidification, we measured the ATP-hydrolytic activity of the V-ATPase in purified lysosomes enriched from PHEV- or mock-infected N2a cells at 48 hpi. The results showed that the ATP hydrolysis rate in lysosomes of PHEV-infected cells, monitored by the release of inorganic phosphate, was strikingly increased compared with that of mock-infected cells (Fig. 6A). Next, we measured the rate of proton translocation into the lysosomal lumen using a pH-sensitive fluorescent probe, 9-amino-6-chloro-2-methoxyacridine (ACMA) (17), which quenches its fluorescence and avoids its permeation through the membrane (Fig. 6B). We observed the rate of decreased fluorescence was higher in lysosomes of PHEV-infected cells than in that of mock-infected cells (Fig. 6C). These results suggest that PHEV infection leads to the enhancement of lysosomal V-ATPase activity.

**Fig 7:**
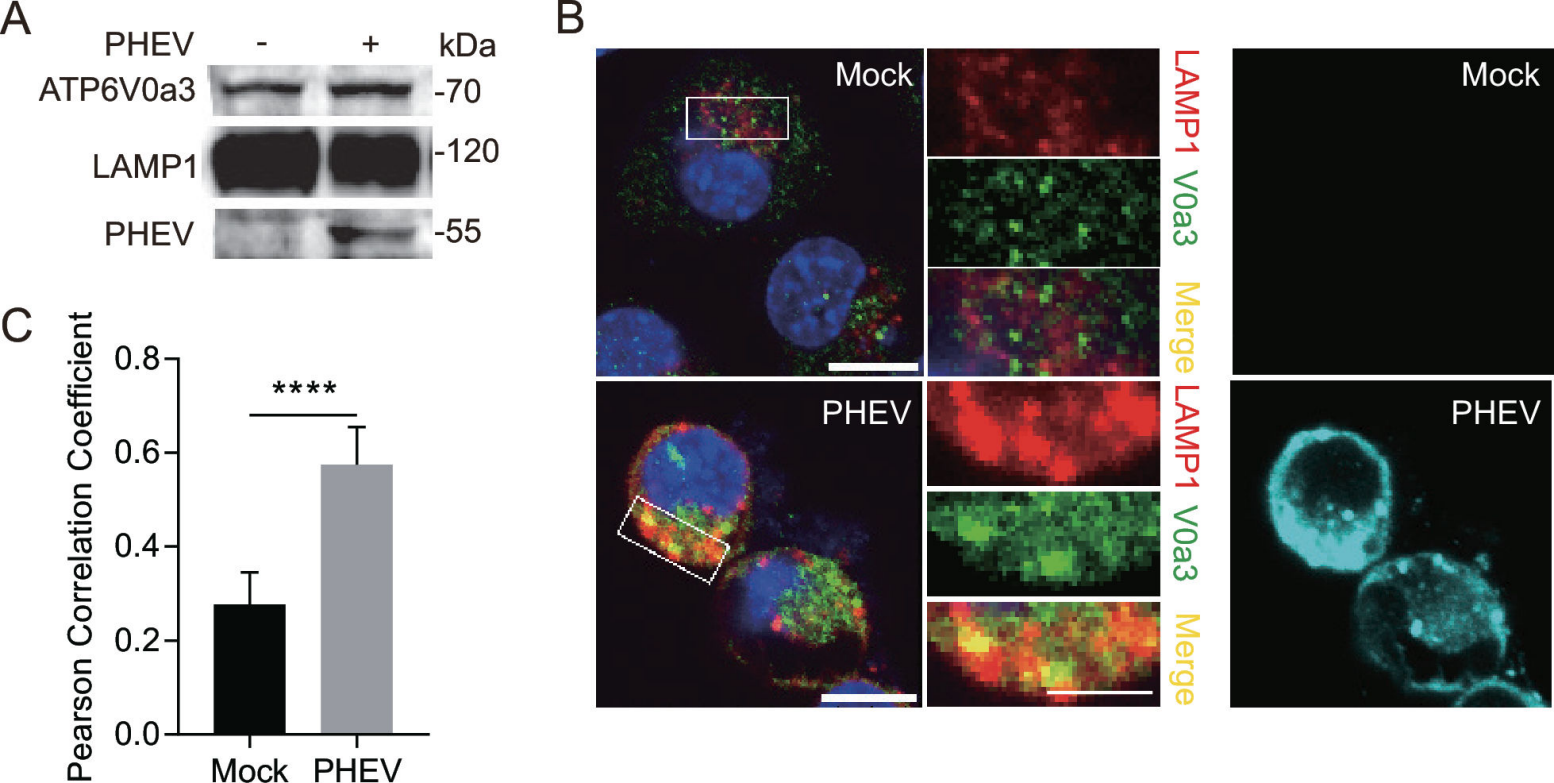
The expression and distribution of V0a3 in PHEV-hijacked lysosomes at 48 hpi. (**A**) The protein levels of V0a3 in mock- and PHEV-hijacked purified lysosomes. (**B**) Colocalization of V0a3 and LAMP1. Mock- or PHEV-infected cells were immunostained with anti-LAMP1 (red), anti-V0a3 (green), and anti-PHEV (teal) antibodies. Scale bar, 10 µm. (**C**) The Pearson’s correlation coefficients of the images between LAMP1 and V0a3 were analyzed by using Image J. Representative blots and images are shown. Data are shown as mean ± SD. *P* values were considered significant when *P* < 0.05 and denoted as, ****, *P* < 0.0001, ns, not significant.

**Fig 8:**
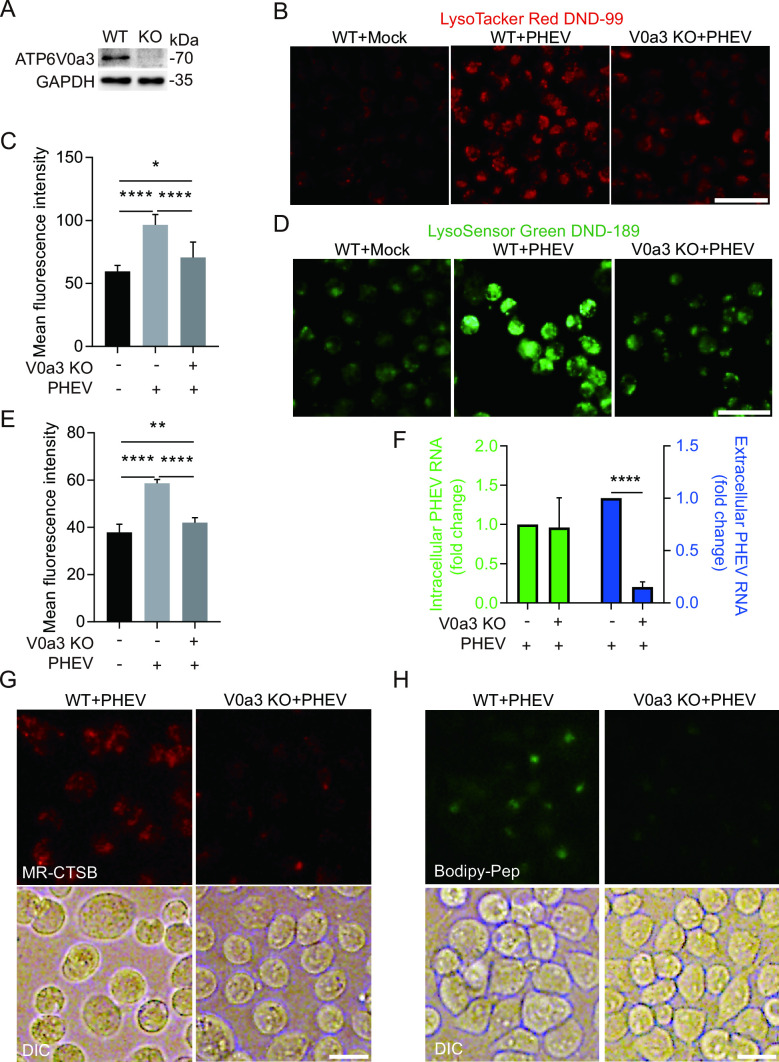
PHEV egress dependents on V0a3-mediated lysosomal acidification (**A**) Determination of V0a3 knockout efficiency using western blotting. (**B**) LysoTracker Red DND-99 staining of mock- or PHEV-infected WT or V0a3 KO cells at 48 hpi. Scale bar, 50 µm. (**C**) Quantification of LysoTracker Red DND-99 fluorescence intensity in mock- or PHEV-infected WT or V0a3 KO cells. (**D**) LysoSensor Green DND-189 staining of mock- or PHEV-infected WT or V0a3 KO cells at 48 hpi. Scale bar, 50 µm. (**E**) Quantification of LysoSensor Green DND-189 intensity in mock- or PHEV-infected WT or V0a3 KO cells. (**F**) The PHEV N genomic RNA was determined using qPCR in PHEV-infected WT or V0a3 KO cells at 48 hpi. The data were normalized to the PHEV-infected WT cells. (**G and H**) Live imaging of Magic Red and Bodipy-FL-pepstatin A dye in PHEV-infected WT or V0a3 KO cells at 48 hpi, which were used as indirect indicators of CTSB and CTSD enzyme activities, respectively. Representative blots and images are shown. Data are shown as mean ± SD. *P* values were considered significant when *P* < 0.05 and denoted as, *, *P* < 0.05, **, *P* < 0.01, and ****, *P* < 0.0001.

The structural diversity of V-ATPase generated by different combinations of subunit isoforms enables it to play diverse physiological roles in mammalian cells (18). To further explore the role of V-ATPase in lysosomal acidity of PHEV-infected cells, the protein level of each V-ATPase subunit in whole-cell lysates was investigated, and the results showed that PHEV infection led to abnormal expression of various V-ATPase subunits (Fig. 6D and E). Especially, PHEV infection not only induced higher expression levels of V0a3 which could facilitate H^+^ transport to lysosomes (19) but also increased V0a3 protein level in lysosomal fractions and led to the enhancement colocalization of V0a3 and LAMP1 (Fig. 7A through C), indicating that V-ATPase subunit V0a3 might play a vital role in lysosomal acidity of PHEV-infected cells by recruiting to the lysosomal membrane to regulate lysosomal acidification. Furthermore, we constructed V0a3-knockout N2a cells (V0a3 KO cells) using the CRISPR/Cas9 system (Fig. 8A). Then it was observed that both the mean fluorescence intensity of LysoTracker and LysoSensor were remarkably decreased in PHEV-infected V0a3 KO cells compared to that in PHEV-infected WT cells at 48 hpi, while they were still significantly increased compared to that in mock-infected WT cells (Fig. 8B through E), indicating that V0a3-knockout could inhibit PHEV enhancing lysosome acidity, and another V-ATPase subunit also contributes to PHEV-induced lysosomal acidification. Moreover, V0a3-knockout could also inhibit PHEV enhancing the activities of CTSD and CTSB and virus release (Fig. 8F through H), suggesting that the PHEV release mechanism involves the disorders of V0a3-dependent lysosomal acidification.

### CID1067700 inhibits PHEV neural transmission in the CNS

It has been reported that βCoVs, such as SARS-CoV-2 and MHV, hijacked lysosomes, the cell’s trash disposal system, to exit cells, and the altered lysosomal function of βCoV-infected cells might result in the perturbation of antigen presentation and lead to altered immune responses (6, 7). This work highlights that targeting regulators of lysosomal trafficking and biogenesis, such as Arl8b and Rab7, may be the potential strategies to stop transmission of the virus that causes βCoVs disease, for instance, COVID-19. However, further research is needed to shed more light on the βCoV using lysosomes for spreading through the body.

**Fig 9:**
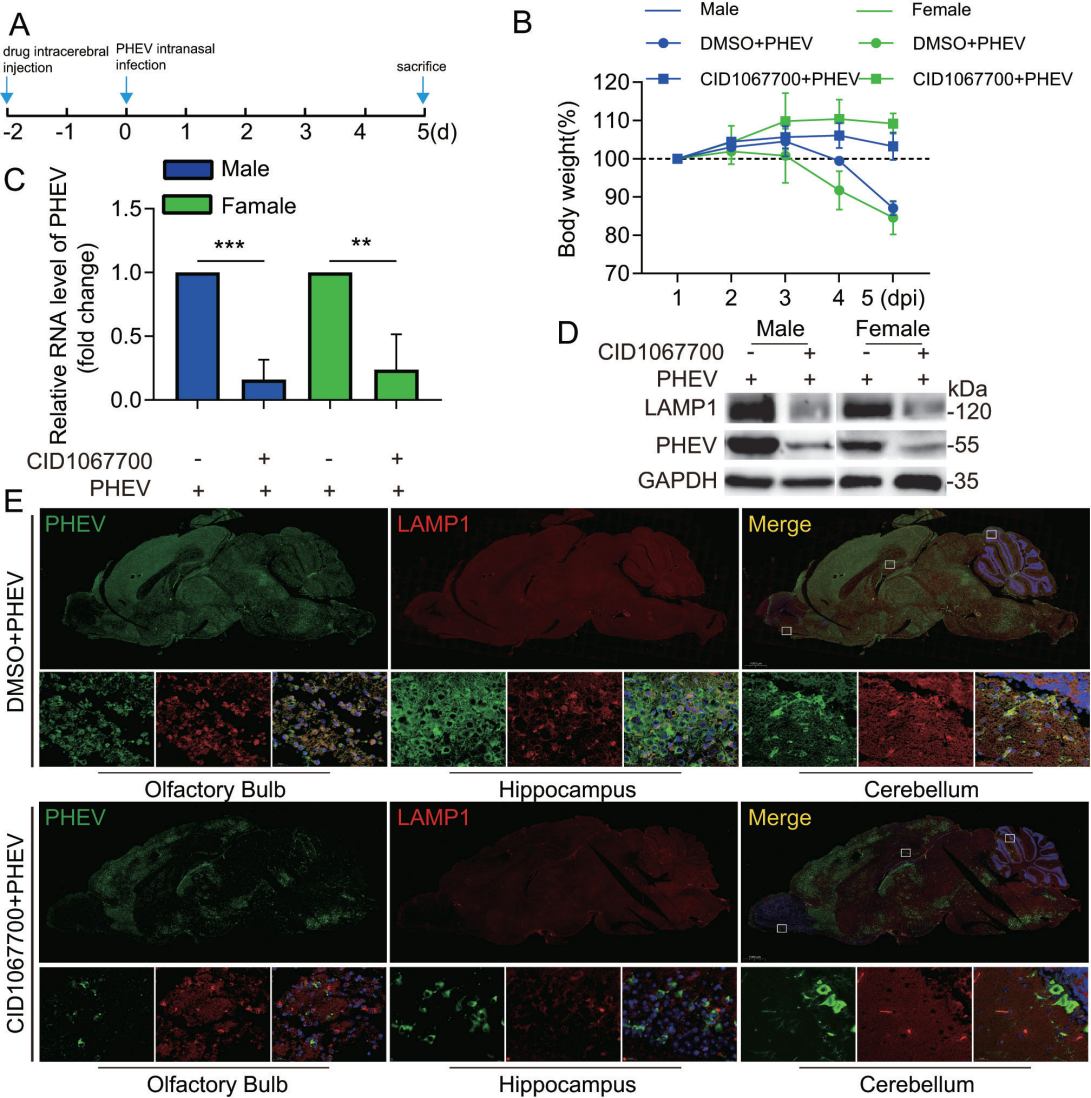
CID1067700 inhibits PHEV neural transmission in CNS. (**A**) Schematic diagrams illustrating the experimental design for time-of-drug treatment and sacrifice experiments. (**B**) Mice were monitored daily for body weight change in different groups. (**C**) PHEV N genomic RNA levels in DMSO- or CID1067700-treated PHEV-infected mice. (**D**) PHEV N protein and LAMP1 levels in DMSO- or CID1067700-treated PHEV-infected mice. (**E**)Colocalization of PHEV and LAMP1 in mice brain sections. DMSO- or CID1067700-treated PHEV-infected brain sections were immunostained with anti-LAMP1 (red) and anti-PHEV (green) antibodies. Representative blots and images are shown. Data are shown as mean ± SD. *P* values were considered significant when *P* < 0.05 and denoted as, **, *P* < 0.01 and ***, *P* < 0.001.

The researchers have employed PHEV-permissive mice or rats as model systems to study CNS pathology associated with PHEV-induced encephalitis (20). In addition, CID1067700, a late endosome GTPase Rab7 receptor antagonist, has been shown to inhibit lysosome numbers in animal brains (21). Thus, to further investigate the effect of lysosomes on PHEV transmission in CNS, 4-week-old BALB/c mice were inoculated by the intracerebral (i.c.) route with DMSO or CID1067700 and intranasally inoculated with PHEV after 2 days, and these mice were monitored daily for body weight change and clinical symptoms (Fig. 9A). The PHEV-infected female or male mice pretreated with DMSO exhibited a substantial decrease in body weight at 4 days post-infection (dpi), while the onset of weight loss was delayed in PHEV-infected female or male mice pretreated with CID1067700 (Fig. 9B). In addition, the PHEV-infected female or male mice pretreated with DMSO began to display typical neurological symptoms at 3 dpi, including generalized muscle tremors and movements of the front and hind feet similar to piano playing, while PHEV-infected female or male mice pretreated with CID1067700 displayed mild or moderate neurological symptoms (Mov. 1). Subsequently, PHEV-infected mice were sacrificed at 5 dpi and then the collected brains were used for qPCR analysis, western blotting analysis, immunofluorescence staining, or histopathological examination to evaluate lysosome biogenesis, virus replication, or histological changes. Results showed that CID1067700 treatment effectively downregulated the protein levels of LAMP1 and markedly reduced PHEV N protein and RNA expression in the brain (Fig. 9C and D), indicating that CID1067700 could weaken PHEV replication by inhibiting lysosomal biogenesis. The number of PHEV-positive cells was significantly decreased in different brain regions (e.g., the olfactory bulb, the hippocampus, and the cerebellum), with a remarkable decrease in lysosome numbers (Fig. 9E). In addition, histological assessment of the brain revealed that CID1067700 attenuates PHEV-induced damage to the brain, including degenerated shrunken neurons with pyknotic nuclei, neuronal necrosis, and sizeable inflammatory cell infiltration (Fig. S4). Taken together, these results indicate that lysosome plays a critical role in PHEV neural transmission and CNS damage caused by virus infection.

## DISCUSSION

Understanding the life cycle of CoV might greatly facilitate future discoveries of more effective therapeutics to treat and prevent CoV-induced illnesses. It has been assumed that CoV hijacks vesicles of the biosynthetic secretory pathway to support viral transport to the plasma membrane and subsequent egress, as is true for other enveloped RNA viruses, such as hepatitis C virus, dengue virus, and West Nile virus (22, 23). However, our findings and results of previous studies indicate that βCoVs, such as PHEV, SARS-CoV-2, and MHV, employ a lysosome-based egress pathway rather than a biosynthetic secretory egress pathway (6). Meanwhile, Saraste et al. have reported evidence indicating that Rab11-positive recycling endosomes may function as transport carriers during the cellular exit of avian infectious bronchitis virus, a γCoV (24). Thus, conflicting findings concerning the roles of secretory compartments in CoV egress suggest that CoVs belonging to different genera may exit cells *via* different mechanisms.

**Fig 10:**
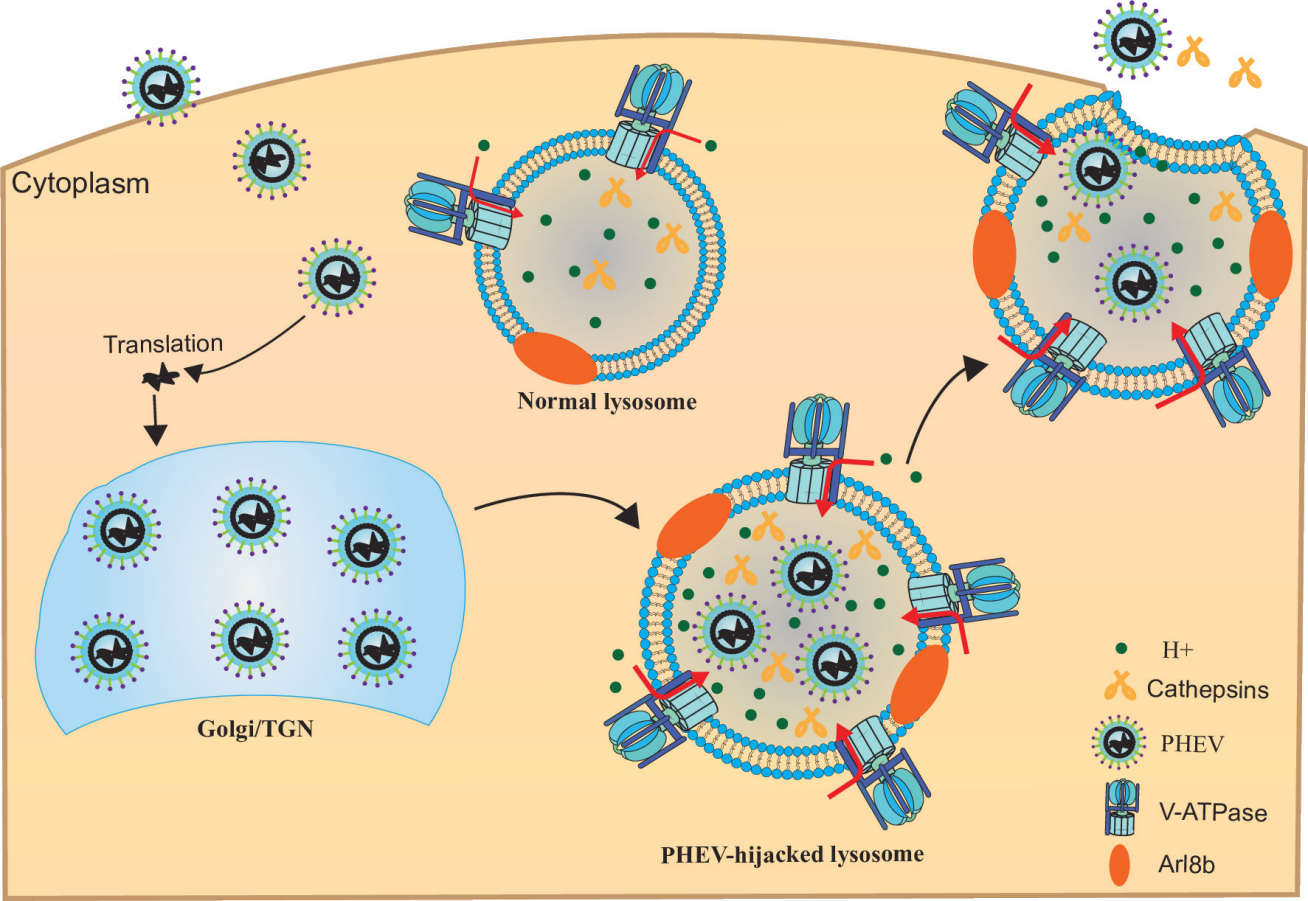
Model of PHEV egress *via* lysosomal exocytic pathway. PHEV promotes Arl8b-dependent on lysosomal exocytosis-mediated viral egress. In addition, PHEV enhances V-ATPase assembly at lysosomes, resulting in increased lysosomal acidity by facilitating H^+^ transport to lysosomes and PHEV egress depends on V-ATPase-dependent lysosomal acidification.

The potential of βCoVs to mutate renders them a constant threat to humans and animals. βCoVs mostly affect the respiratory, intestinal tracts, and CNS of both humans and animals. Although SARS-CoV-2 is considered a respiratory pathogen, of great concern is the results of recent reports that have revealed that it shares neurotropic and neuroinvasive properties with almost all other known βCoVs (25, 26). PHEV can cause neurological disorders, vomiting and wasting disease, or influenza-like illness in pigs (27). Replication of βCoV begins with virus attachment to host cells that are initiated by interactions between viral structural proteins, known as spike (S) proteins, and specific host cell receptors, with different βCoVs utilizing different virus attachment mechanisms. Thus, the S-protein-cell receptor interaction, by governing the tissue tropism of a virus, is the primary determinant of whether a given CoV species can infect a particular host species. For example, SARS-CoV-2, MHV, and PHEV use angiotensin converting-enzyme 2 (ACE2) (28, 29), carcinoembryonic antigen cell adhesion molecule (30), and cell-surface glycans (e.g., sialic acid and heparan sulfate) (31), respectively, as host cell receptors for virus attachment. To generate progeny virus particles, after a βCoV particle binds to its host cell receptor, it must enter the cell to initiate downstream steps in the viral lifecycle that culminate in viral assembly and uptake by lysosomes and viral egress from cells *via* exocytosis. Here, PHEV within cell bodies of infected sensory neurons was observed to bud from the endoplasmic reticulum–Golgi intermediate compartments and then assemble to form either individual virus particles within small vesicles or multiple virus particles within large vesicles (8, 32, 33). Meanwhile, viral nonstructural proteins were found to assemble on endoplasmic reticulum-derived membranes and participate in viral RNA replication. Thereafter, PHEV was observed to traffic to lysosomes and exit cells *via* an Arl8b-dependent lysosomal exocytosis mechanism resembling that used by MHV and SARS-CoV-2 (3). Moreover, PHEV egress depends on the viral hijacking of actively or passively acidified lysosomes instead of deacidified lysosomes, the latter of which are associated with SARS-CoV-2 and MHV egress (Fig. 10). Thus, different βCoVs hijacked-lysosomes exhibit alternative forms of dysfunction for virus release. Additionally, more should be done in the future to understand the mechanism of active and passive lysosome deacidification or acidification in βCoV-infected cells to verify the effects of reversing lysosomal pH on βCoV egress.

Koya et al. have found that distinct motifs in SARS-CoV-2 envelope protein, as a transmembrane protein, participate in lysosomal deacidification in virus-infected host cells (34). However, the mechanisms underlying SARS-CoV-2- or MHV-induced deacidification still remain largely unknown. It is uncertain whether SARS-CoV-2- or MHV-induced lysosomal deacidification is directly caused by the disturbed proton pump (35). Lysosome pH gradients are primarily maintained by V-ATPase, which pumps protons into lysosomal lumen by consuming ATP. V-ATPase dysfunction can lead to the disorder of lysosomal acidification. The V-ATPase complex pumps protons into the lumen of lysosome coupled with ATP hydrolysis in all types of cells and is composed of multiple core subunits: the cytosolic V1 domain includes eight different subunits (A, B, C, D, E, F, G, and H) and the integral V0 domain contains six different subunits (a, c, c’, c”, d, and e) (36). The different expression levels of each subunit can lead to different permutations of subunit structure in individual proton pumps and unique subunit identities of pumps at different locations, which may allow the regulation of V-ATPase in a cell-type and subcellular compartment-specific manner (36). Taken together, the βCoVs induce the changes in lysosomal pH by affecting V-ATPase activity *via* altering the expression and distribution of V-ATPase subunits, warranting further study.

The term PHEV-induced-lysosomal exocytosis refers to the regulated extracellular release of the contents of PHEV-hijacked lysosomes. During this process of lysosomal exocytosis, lysosomes can migrate to the cell surface proximity and fuse with the plasma membrane, releasing their contents extracellularly. It has been reported that V-ATPase subunit ATP6AP1 regulates lysosomal trafficking and protease exocytosis in osteoclast-mediated bone resorption, and V-ATPase subunit V0a3 not only plays a critical role in V-ATPase activity but also is essential for anterograde trafficking of secretory lysosomes (37, 38). These findings indicate that V-ATPase subunit expression and distribution are involved lysosomal exocytosis. Our results have demonstrated that PHEV infection might regulate V-ATPase subunits such as V0a3 and V0a1 expression and enhance V-ATPase activity by altering the expression and distribution of V-ATPase subunit V0a3, but we still need to study in detail correlation between V0a1- and V0a3-mediated lysosomal acidification and exocytosis after PHEV infection. Additionally, we still need to focus on the role of other V-ATPase subunits like ATP6AP1 in PHEV replication, which may also contribute to PHEV-induced lysosome acidification or exocytosis.

Normally, the acidic lysosomal environment helps destroy invading viruses and other pathogens before they can leave cells to start another infection cycle. An abnormal rise or fall in lysosomal pH has far-ranging effects on lysosomal degradation—strongly inhibiting hydrolases with the most acidic pH optima, but also potentially elevating activities of other hydrolases with pH optima closer to or away from neutral (39). Interestingly, PHEV infection led to active or passive acidification of lysosomes that could enhance PHEV egress and increase the activation of CTSD and CTSB, while SARS-CoV-2 or MHV hijacked lysosomes by deacidifying their internal environments and led to the inactivation of lysosomal degradation enzymes (6, 7). To prevent effective lysosomal clearance of PHEV, this non-lytic viral release process may depend on associated lysosomal dysfunction. Indeed, we have found that in order to avoid lysosomal degradation of progeny virus, PHEV infection can lead to lysosome disorders and showed that the specific mechanism of lysosome dysfunction is related to progranulin expression deficiency (4). In addition, by a comprehensive proteomic study of lysosomal fractions enriched from mock and PHEV-infected nerve cells, we have screened other proteins participating in PHEV-induced lysosomal dysfunction (data not shown), and the detailed mechanisms of them remain unexplored, warranting further study.

In addition, this is the first study to evaluate βCoV using lysosome for spreading through the body, and we found that lysosome played a critical role in PHEV neural transmission and brain damage caused by virus infection in CNS. It’s worth noting that Li et al. have reported that the infectious transmission of PHEV is likely through a mixture of virus-modified exosomes, which mediate the transfer of immunostimulatory cargo to uninfected neuroimmune cells (40), and the results of our previous paper have shown that PHEV could also induce apoptosis or programmed cell death of host cells for virus replication (41), suggesting that PHEV may use a variety of ways or complex ways for progeny virus spread *in vitro* or *in vivo*.

Collectively, the results of this study demonstrated that lysosomes played an important role in PHEV release or transmission *in vitro* or *in vivo*. These findings show unique interactions between lysosomes and different βCoVs, which facilitate the discovery of new therapeutic strategies to reverse virally induced lysosomal dysfunction to mitigate βCoV infection and slow virus spread.

## MATERIALS AND METHODS

### Cell lines and viruses

N2a and HT22 cells were cultured in the complete DMEM (Gbico) supplemented with 10% fetal bovine serum (FBS) and 1% penicillin/streptomycin in the cell incubator and maintained at 37°C in 5% CO_2_.

The PHEV strain used in this study was PHEV CC14 (GenBank accession number MF083115.1), which was maintained by our research group (4). The cells were infected with PHEV 10^6.80^ [50% median tissue culture infectious dose (TCID_50_) /0.1 mL] in cell cultures with 2% FBS for 1 h. Following the 1 h absorption period, infected cells were incubated in the complete DMEM at 37 °C in 5% CO_2_ for the relevant time in conformity with the experimental requirements.

### Animal experiments

Four-week-old BALB/c mice (male or female) were obtained from the Laboratory Animal Center of Jilin University. For *in vivo* treatment, CID1067700 was diluted with DMSO (stock concentration 40 mM). The BALB/c mice were inoculated by the i.c. route with 16 mg/kg CID1067700. After 2 days, the mice were intranasally inoculated with 50 µL PHEV (10^6.80^ TCID_50_/0.1 mL). Subsequently, PHEV-infected mice at 5 dpi were sacrificed by CO_2_ asphyxiation according to animal handling guidelines. After sacrifice, the brain of each mouse was surgically excised and placed on an ice pad for further testing.

### Antibodies, reagents, and plasmids

The mouse anti-PHEV-N monoclonal antibody was developed in our laboratory. The following primary antibodies were used: anti-CTSD antibody (Abcam, ab75852), anti-CTSB antibody (Abcam, ab214428), anti-CD107a antibody (BD Biosciences, 553792), anti-LC3 antibody (Sigma-Aldrich, L8918), anti-ATP6V0a3 antibody (Proteintech, 12649–1-AP), anti-ATP6V0a1 (Santa Cruz Biotechnology, sc-374475), anti-ATP6V1A antibody (Abcam, ab199326), anti-ATP6V1B1 + ATP6V1B2 antibody (Abcam, ab200839), anti-ATP6V1D antibody (Abcam, ab157458), anti-glyceraldehyde-3-phosphate dehydrogenase (GAPDH) antibody (Proteintech, 60004–1-Ig), anti-ARL8B antibody (Affinity Biosciences, DF12350), anti-RCAS1 antibody (Cell Signaling Technology, 12290), and anti-ERp72 antibody (Proteintech, 14712–1-AP). The secondary antibodies used for western blotting were horseradish peroxidase (HRP)-conjugated anti-mouse, anti-rat, or anti-rabbit IgG (Proteintech). The secondary antibodies used for immunofluorescence assay (IFA) were Alexa Fluor 488-conjugated goat anti-mouse/rabbit IgG, Alexa Fluor 594-conjugated goat anti-mouse/rabbit IgG, and Alexa Fluor 647-conjugated goat anti-mouse/rabbit IgG, all of which were purchased from Cell Signaling Technology. Alexa Fluor 594-conjugated goat anti-rat IgG was purchased from Abam. 12 nm colloidal gold-affinipure goat anti-rat IgG was purchased from Jackson ImmunoResearch. CID1067700, Chloroquine, Concanamycin A, and Staurosporine were purchased from MCE. The V-ATPase inhibitor Bafilomycin A1 was purchased from Cell Signaling Technology. ACMA, Lysotracker Red DND-99, and Lysosensor Green DND-189 were purchased from ThermoFisher Scientific. Hoechst 33342 and Propidium Iodide were purchased from Beyotime.

Plasmids LAMP1-mCherry and GFP-Arl8b were constructed using conventional cloning techniques. The target fragments were amplified by PCR and were then cloned into the pCDNA3.1-mCherry or pCMV-C-EGFP vector, respectively. All constructs were validated by DNA sequencing.

### DNA transfections and RNA interference

DNA transfection was carried out with Lipofectamine 3000 transfection reagent (ThermoFisher Scientific) according to manufacturer instructions. Briefly, the plasmid DNA and transfection reagent were incubated in a separate tube for 15 min. The plasmid DNA/Lipofectamine 3,000 mixture was pipetted slowly onto the culture of cells. For the RNA interference assay, siRNAs against Arl8b were designed based on the full-length mRNA sequences (accession number NM_026011.3). The sequences were as follows: siArl8b1, 5′-GGUACUGCCGAGGAGUCAATT-3′; siArl8b2, 5′-GGAAAGUAACUAAAGGCAATT-3′. A siRNA with a control sequence irrelevant to all known genes was also designed and synthesized. N2a cells were transfected with the appropriate siRNA by using GP-transfect-Mate transfection reagent (GenePharma) according to the manufacturer’s instructions. Subsequent experiments were performed 24 h after transfection.

### qPCR and western blotting

Total RNA was extracted using TRIzol reagent (Invitrogen), and reverse transcription was performed using PrimeScript reverse transcriptase (TaKaRa). PHEV N gene RNA was detected as previously described using primer sequences that targeted the N gene of PHEV (5). qPCR was conducted with 2× SYBR qPCR master mix (Biomake) on a CFX96 Touch real-time PCR detection system (Bio-Rad), with GAPDH as a control for normalization. For western blotting, cells were lysed in RIPA buffer then lysates were loaded onto SDS-PAGE gels and subjected to electrophoresis. Next, separated proteins in gels were transferred to PVDF membranes (Millipore) that were probed with appropriate antibodies. Western blotting signals were analyzed using Image J software.

### Immunofluorescence staining and confocal microscopy

PHEV- or mock-infected cells grown on glass coverslips were fixed in 4% paraformaldehyde for 15 min; then washed three times with phosphate buffer solution (PBS), permeabilized, and blocked-in blocking buffer for 1 h. Primary antibodies diluted in blocking buffer were applied to the cells overnight at 4°C. Coverslips were washed three times with PBS. Secondary antibodies and Hoechst stain diluted in blocking buffer were applied to the cells for 1 h at 37°C. For cell surface LAMP1 staining, cells were pre-chilled at 4°C for 20 min and incubated on ice with anti-LAMP1 antibody in PBS for 30 min. After rinsing with chilled PBS, cells were kept on ice and incubated with appropriate secondary antibody in PBS for 30 min. Cells were rinsed with chilled PBS, fixed in chilled 2% paraformaldehyde (PFA) for 5 min, rinsed, and mounted. Coverslips were viewed under the Olympus FV3000 confocal microscope (Olympus). Image J was used for all image analysis including quantification of LAMP1, CTSB, GFP-Arl8b, Lysotracker Red DND-99, etc. It should be noted that during fixation, lysosomal size and area might have been changed, but we always have a control group and an experimental group analyzed at the same time.

### Transmission electron microscopy

N2a cells were infected with PHEV, and the samples were collected to fix in 4% formaldehyde and 0.1% glutaraldehyde for 2 h for thin sectioning and observed using TEM as previously described (33).

### Immunoelectron microscopy

N2a cells were infected with PHEV and fixed in 4% formaldehyde and 0.1% glutaraldehyde for 2 h. The samples were conducted in thin sections. Immunogold labeling was carried out on sections with anti-LAMP1 (1:20, rat) antibodies. Then the sections were incubated with 12 nm gold (1:10) and embedded in 1% glutaraldehyde. Sections were examined with a transmission electron microscope.

### Lysotracker and Lysosensor pH measurements

Mock- and PHEV-infected cells were incubated with Lysotracker Red DND-99 (100 nM) according to the manufacturer’s instructions. For Lysosomal pH measurement, we used Lysosensor Green DND-189 to treat cells, which exhibit increasing fluorescent intensity in response to acidification. A standard pH curve was constructed by incubating cells with potassium buffers of known pH containing 10 µg/mL Nigericin (MCE). Mock- and PHEV-infected cells were incubated with Lysosensor Green DND-189 for 1.5 h and determined fluorescence intensity. The standard curve was used to convert the fluorescence values to pH values.

### Lysosomal enzyme activity assay

Mock- and PHEV-infected cells were lysed in chilled CD or CB cell lysis buffer and incubated cells on ice for 10 min. An amount of 100 ng cell lysate was analyzed according to the manufacturer’s instructions of the CTSD and CTSB activity assay kit (Abcam). Then the lysosomal enzyme activity was measured with a fluorescent microplate reader.

In addition, to further investigate CTSB activity in mock- or PHEV-infected cells, MagicRed-CTSB (Immunochemistry Technologies) was added to cells at the concentration suggested by the company (1:260) and then incubated for 30 min. To assess levels of CTSD activity, Bodipy-FL-pepstatin A (Life Technologies) was added to the cells in a final concentration of 1 µg/mL for 60 min. After washing the cells with PBS, a new medium was added for live imaging by confocal microscopy.

### Generation of knockout cell lines by CRISPR/Cas9

CRISPR–Cas9 technique was used to knock out V0a3. The single guide RNA sequence (5′-TGAGCGCCTTCCAGAGACGC-3′) targeting the mouse V0a3 gene (accession number AF218253.1) was cloned into the pSpCas9(BB)−2A-Puro (PX459) vector. Then the constructed plasmid was introduced into N2a cells using Lipofectamine 3,000 transfection reagent. After incubation for 48 h, stable cell lines were established after 6 days of puromycin (4 µg/mL) selection, and knockdown of the target gene was confirmed by western blotting analysis.

### Measurement of V-ATPase activity

The procedure described by Lee et al. was modified and used for measuring ATP hydrolysis (42). In brief, lysosome-enriched fractions (32.5 µg total protein) were mixed with 3 (vol) of Buffer A (100 mM MES–Tris buffer, 80 mM KCl, 6 mM MgCl_2_, 150 mM NaCl, and pH 7.0) and incubated at 37°C for 5 min. After incubation, the reaction was started by the addition of 5 mM ATP and incubated for 20 min at 37°C and then added the addition of 2 mL of molybdate solution [2% (vol/vol) H_2_SO_4_, 0.5% (wt/vol) ammonium molybdate, and 0.5% (wt/vol) SDS] and 0.2 mL of ascorbic acid [2% (wt/vol)] to develop for 5 min at 37°C. Control samples were measured in the presence of the V-ATPase inhibitor BafA1 (1 µM), and the experimental values were subtracted accordingly. Absorbance was measured at 750 nm, and solutions of KH_2_PO_4_ were used to generate a standard curve.

### Proton translocation assay

Proton transport activity into the lumen of isolated lysosomes was measured by fluorescence quenching of ACMA in the presence or absence of 1 µM BafA1. Lysosomes (25 µg) were added to a cuvette containing 2 mL of reaction buffer [10 mM BisTrisPropene (BTP)-MES, pH 7, 25 mM KCl, 2 mM MgSO4, 10% glycerol, and 2 µM ACMA]. The reaction was started by the addition of 2 mM ATP, a measurement (ex412/em480) taken every 60 seconds for 600 seconds with a fluorescent microplate reader.

### Trypan blue and propidium iodide staining for plasma membrane permeability

Mock- and PHEV-infected cells were rinsed in PBS. Then cells were incubated with 0.4% trypan blue stain for 5 min at room temperature. Trypan blue was removed, and cells were imaged by an optical microscope (Olympus). Mock- and PHEV-infected cells were rinsed in PBS. Then cells were incubated with propidium iodide diluted by DMEM for 20 min at room temperature. Propidium iodide was removed, and cells were imaged by a fluorescence microscope (Olympus). The group of staurosporine treatment was viewed as a positive control in the above experiments.

### Lysosome purification

Lysosomes were then isolated from cells using the Minute Lysosome Isolation Kit (Invent Biotechnologies) based on the spin-column method. Briefly, mocked or PHEV-infected cells were suspended in Buffer A to incubate on ice for 10 min and immediately transferred to the filter cartridge to centrifuge at 16,000 × *g* for 30 seconds. Then discarded the filter, and differential centrifugation (2,000 × *g* for 3 min, 11,000 × *g* for 15 min, and 16,000 × *g* for 30 min) was used to obtain the pellet. The pellet was resuspended in 200 µL cold Buffer A and 100 µL Buffer B to incubate the tube on ice for 30 min and centrifuged at 11,000 × *g* for 10 min. Finally, the pellet was resuspended in 100 µL PBS to obtain the highly enriched lysosome fraction.

### Statistical analysis

Graphics and statistical tests were performed using GraphPad Prism v8.0 software (GraphPad Software, San Diego, CA). Data are presented as the means ± SD. Statistical analyses were performed on data from triplicate experiments by using Student’s *t* test. In all cases, a *P* value of ≤ 0.05 was considered statistically significant.

## Data Availability

Underlying data and the accession numbers are available in the main text. All other raw data will be shared upon reasonable request.

## SUPPLEMENTAL MATERIAL

Fig. S1 jvi.01338-23-s0001.tif Kinetics of PHEV replication and release in N2a and HT22 cells, respectively.

Fig. S2 jvi.01338-23-s0002.eps PHEV hijacks lysosomes and uses Arl8b-dependent lysosomal exocytosis for egress.

Fig. S3 jvi.01338-23-s0003.eps The effect of CQ treatment on PHEV release based on the experiment design.

Fig. S4 jvi.01338-23-s0004.tif CID1067700 attenuates PHEV-induced damage to the brain.

Supplemental Legends jvi.01338-23-s0005.docx Legends for Fig. S1 to S4 and Movie S1.

Movie S1 jvi.01338-23-s0006.mp4 The PHEV-infected mice pretreated with DMSO or CID1067700 display neurological symptoms.

ASM does not own the copyrights to Supplemental Material that may be linked to, or accessed through, an article. The authors have granted ASM a non-exclusive, world-wide license to publish the Supplemental Material files. Please contact the corresponding author directly for reuse.
