# Supplementary figures and images for: The role of lysosomes as intermediates in betacoronavirus PHEV egress from nerve cells
Source: J Virol. 2023 Nov 27;97(12):e01338-23. doi: 10.1128/jvi.01338-23 (PMC10734498; doi:10.1128/jvi.01338-23)

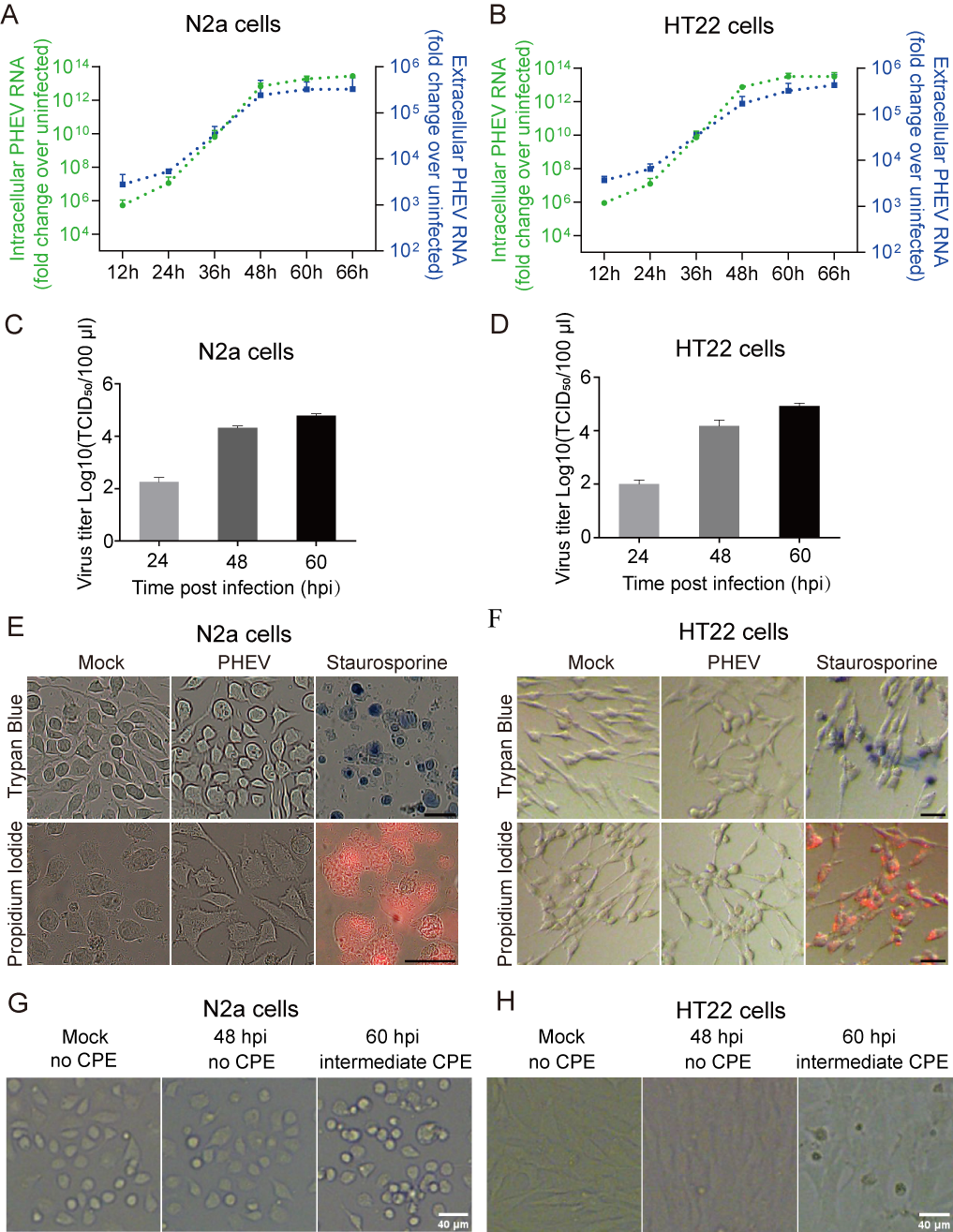

Supplement: Fig. S1 — Kinetics of PHEV replication and release in N2a and HT22 cells, respectively. [file jvi.01338-23-s0001.tif]

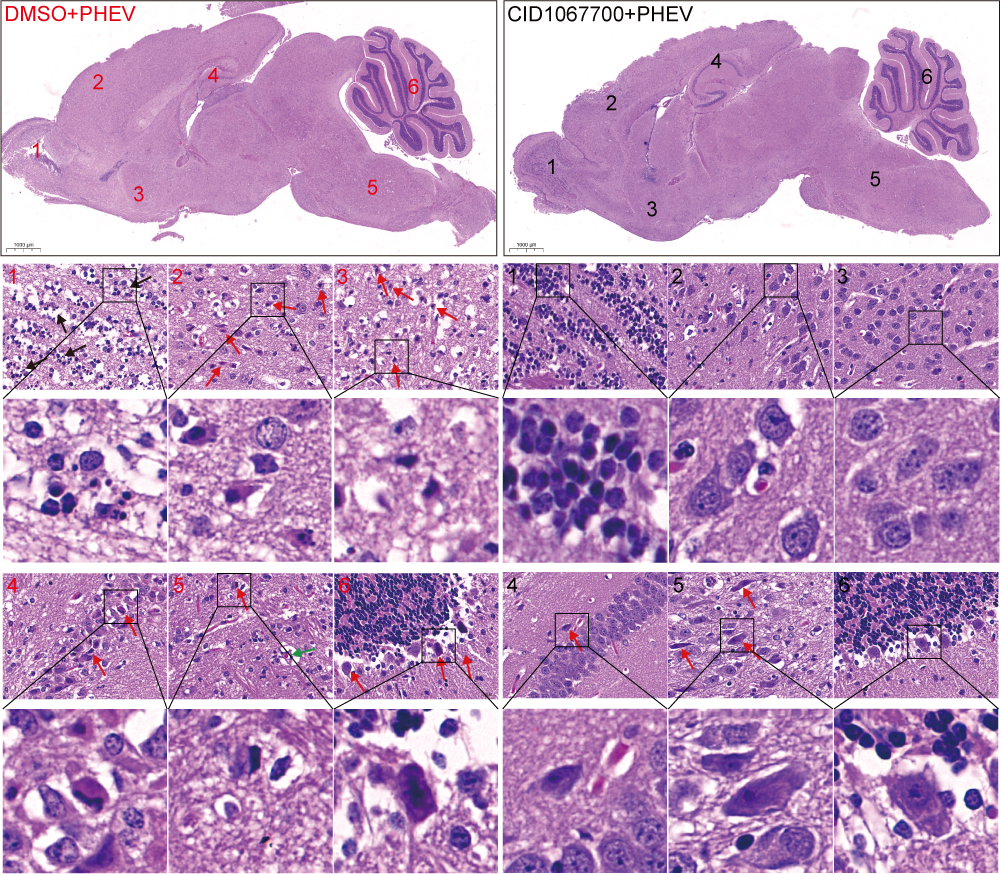

Supplement: Fig. S4 — CID1067700 attenuates PHEV-induced damage to the brain. [file jvi.01338-23-s0004.tif]
