# Supplementary material for: The role of lysosomes as intermediates in betacoronavirus PHEV egress from nerve cells
Source: J Virol. 2023 Nov 27;97(12):e01338-23. doi: 10.1128/jvi.01338-23 (PMC10734498; doi:10.1128/jvi.01338-23)
Supplement: Supplemental Legends — Legends for Fig. S1 to S4 and Movie S1. [file jvi.01338-23-s0005.docx]

**SUPPLEMENTAL MATERIAL**

**Fig S1** Kinetics of PHEV replication and release in N2a and HT22 cells, respectively. (A and B) PHEV N genomic RNA levels quantified by qPCR in cell lysates and extracellular medium in N2a and HT22 cells, respectively. (C and D) Viral titers at different time by TCID_50_ endpoint assay. (E and F) Trypan blue and propidium iodine exclusion were used to detect changes in plasma membrane permeability in PHEV-infected N2a and HT22 cells at 48 hpi, respectively. Staurosporine-treated cells were seen as a positive control of cell membrane rupture. Scale bar, 30 μm. (G and H) Development of CPE was exemplarily shown for PHEV at selected time points. Representative images are shown. Scale bar, 40 μm. Data are shown as mean ± SD.

**Fig S2** PHEV hijacks lysosomes and uses Arl8b-dependent lysosomal exocytosis for egress. (A) Colocalization of endogenous LAMP1 and ERp72 in N2a cells. Mock- and PHEV-infected cells at 48 hpi were immunostained with anti-LAMP1 (red), anti-ERp72 (green) and anti-PHEV (teal) antibodies. Scale bar, 10 μm. (B) Colocalization of RCAS1 and LAMP1 in N2a cells. Mock- and PHEV-infected cells at 48 hpi were immunostained with anti-LAMP1 (red), anti-RCAS1 (green) and anti-PHEV (teal) antibodies. Scale bar, 10 μm. (C) Colocalization of PHEV and LAMP1-mCherry in N2a cells. Mock- and PHEV-infected cells at 48 hpi were immunostained with anti-PHEV (green) antibodies. Scale bar, 10 μm. (D) LAMP1 and anti-PHEV coimmunostaining in DMSO- and CID1067700-treated (20 μM, 24 hpi) cells. PHEV-infected cells were immunostained with anti-LAMP1 (red) and anti-PHEV (green) antibodies. Scale bar, 10 μm. (E) Colocalization of LAMP1 and Arl8b-GFP in N2a cells. Mock- and PHEV-infected cells were immunostained with anti-LAMP1 (red) and anti-PHEV (teal) antibodies. Scale bar, 10 μm.

**Fig S3** The effect of CQ treatment on PHEV release based on the experiment design. (A) The PHEV-infected cells treated with CQ or water were stained with LysoTracker Red DND-99. Scale bar, 20 μm. (B) The PHEV N genomic RNA was determined using qPCR in water- or CQ-treated PHEV-infected N2a cells. The data were normalized to the water-treated PHEV-infected cells. Representative blots and images are shown.

**Fig S4** CID1067700 attenuates PHEV-induced damage to the brain. H&E staining of brain sections from PHEV-infected mice with DMSO or CID1067700 pretreatment (Black arrows: apoptosis/single cell necrosis; Red arrows: shrunken neurons with pyknotic nuclei/nuclear pyknosis, Green arrows: edema).

**SUPPLEMENTAL MOVIE**

**Mov 1** The PHEV-infected mice pretreated with DMSO or CID1067700 display neurological symptoms. The PHEV-infected mice pretreated with DMSO began to display typical neurological symptoms, including generalized muscle tremors and movements of the front and hind feet similar to piano playing, while PHEV-infected female or male mice pretreated with CID1067700 displayed mild or moderate neurological symptoms.
